# Longitudinal relationships between speech perception, phonological skills and reading in children at high‐risk of dyslexia

**DOI:** 10.1111/desc.12723

**Published:** 2018-09-12

**Authors:** Margaret J Snowling, Arne Lervåg, Hannah M Nash, Charles Hulme

**Affiliations:** ^1^ Department of Experimental Psychology University of Oxford Oxford UK; ^2^ Department of Education University of Oslo Oslo Norway; ^3^ Department of Psychology University of Leeds Leeds UK; ^4^ Department of Education University of Oxford Oxford UK

## Abstract

Speech perception deficits are commonly reported in dyslexia but longitudinal evidence that poor speech perception compromises learning to read is scant. We assessed the hypothesis that phonological skills, specifically phoneme awareness and RAN, mediate the relationship between speech perception and reading. We assessed longitudinal predictive relationships between categorical speech perception, phoneme awareness, RAN, language, attention and reading at ages 5½ and 6½ years in 237 children many of whom were at high risk of reading difficulties. Speech perception at 5½ years correlated with language, attention, phoneme awareness and RAN concurrently and was a predictor of reading at 6½ years. There was no significant indirect effect of speech perception on reading via phoneme awareness, suggesting that its effects are separable from those of phoneme awareness. Children classified with dyslexia at 8 years had poorer speech perception than age‐controls at 5½ years and children with language disorders (with or without dyslexia) had more severe difficulties with both speech perception and attention control. Categorical speech perception tasks tap factors extraneous to perception, including decision‐making skills. Further longitudinal studies are needed to unravel the complex relationships between categorical speech perception tasks and measures of reading and language and attention.

**RESEARCH HIGHLIGHTS**

- Speech perception at 5½ years is a predictor of reading at 6½ years and its effects are separable from those of phoneme awareness.
- Language at 5½ years predicts phoneme awareness and shares variance with attention and speech perception.
- Deficits in speech perception are larger in children with language problems than children with reading difficulties but both groups make more errors on easy ‘catch trials’ than controls.

## 1 INTRODUCTION

Developmental theories proposing a causal chain from auditory sensitivity through speech perception to phonological awareness and reading (e.g. Tallal, 1980) provide a framework within which to consider the etiology of reading problems (Zhang & McBride‐Chang, 2010). It is well established that a phonological deficit is a causal risk factor for dyslexia (Snowling & Melby‐Lervåg, 2016) and it has been hypothesized that phonological deficits in dyslexia have their origins in poor speech perception (e.g. Brandt & Rosen, 1980; Godfrey, Syrdal‐Lasky, Millay & Knox, 1981; Werker & Tees, 1987). Evidence for this hypothesis is weak, however (see Schulte‐Körne & Bruder, 2010, for a review). Very few studies have tested children young enough to assess the claim and those that have are small in scale (Boets, Wouters, van Wieringen, & Ghesquière, 2007; Gerrits & de Bree, 2009; Pennala et al., 2010; van Alphen et al., 2004; Vandenwalle, Boets, Ghesquière, & Zink, 2012). Furthermore, although sensitivity to auditory and speech stimuli in infancy measured using ERP tasks is associated with later reading (e.g. Leppänen et al., 2012; Molfese, 2000), how such effects relate to typical behavioural measures of speech perception is uncertain.

The majority of studies investigating the relationship between speech perception and learning to read use categorical perception tasks (e.g. Adlard & Hazan, 1998; Manis et al., 1997; Messaoud‐Galusi, Hazan, & Rosen, 2011; Nittrouer, 1999). In such tasks, a gradual change in the perceptual features of a syllable (e.g. its voice onset time) is perceived as a sudden change from one phonemic category to another. For example, the phonemes /b/ and /p/ differ in voice onset time (the temporal relationship between the onset of laryngeal vibrations and the release of the vocal tract closure, which occurs earlier for /b/ than /p/). Two hypotheses have been assessed in relation to dyslexia. First, that readers with dyslexia may have less clear‐cut perceptual categories for speech than typical readers: they show flatter identification slopes and poorer discrimination around phoneme boundaries. Second, they may be over‐sensitive to changes within categories and exhibit an ‘allophonic’ mode of processing (Noordenbos & Serniclaes, 2015; Serniclaes, van Heghe, Mousty, Carré, & Sprenger‐Charolles, 2004). In either case, poor phonemic categorization can be expected to compromise the development of phonological representations, with knock‐on effects for phoneme awareness and reading, as is seen in dyslexia. However, since studies on speech perception in dyslexia have typically used samples of older children or adults, an alternative view is that learning to read refines speech perception and in children with reading difficulties this refinement of perceptual categories as a consequence of reading is compromised (Horlyck, Reid, & Burnham, 2012).

A set of factors relating to participant characteristics further complicate the interpretation of evidence on speech perception in dyslexia. Most existing studies have used case‐control designs that are vulnerable to bias if the participants are not screened for comorbid disorders. Joanisse, Manis, Keating, and Seidenberg (2000) were the first to observe that speech perception deficits only affected a subgroup of children with dyslexia, specifically, those with co‐occurring language difficulties. Following on from this, Robertson, Joanisse, Desroches, and Ng (2009) showed that children with dyslexia without comorbid specific language impairment (SLI) showed only marginal difficulties on a categorical speech perception task while children with SLI showed clear deficits. In a similar vein, Rosen (2003) hypothesized that the attentional demands of categorical speech perception tasks are such that children with co‐occurring attentional difficulties will perform poorly on these tasks. Messaoud et al. (2011) tested this hypothesis by examining performance in a categorical perception task on the clear tokens at the endpoints of a continuum varying in voice onset time (‘bee–pea’). Children with dyslexia more often mislabelled these ‘easy’ trials than controls and the tendency to do so increased as the experiment progressed, suggesting fatigue and waning of attention. In short, when investigating speech perception deficits as putative causal risk factors for dyslexia it is important to take the precaution of controlling for difficulties in language and attention which are common in children at risk of reading difficulties.

Notwithstanding these complications, the investigation of speech perception deficits in dyslexia follows from the assumption that the development of phonological representations plays a causal role in learning to read (e.g. Goswami, 2015). Tests of the causal hypothesis that difficulties in speech perception affect reading via effects on phonological skills have been made by McBride‐Chang and colleagues (McBride‐Chang,^1996^; McBride‐Chang, Wagner, & Chang, 1997; Zhang & McBride‐Chang, 2014). In a cross‐sectional study, McBride‐Chang (1996) tested third and fourth graders on measures of categorical speech perception and phonological skills (tasks tapping phoneme awareness, verbal short‐term memory and rapid naming). Concurrently, speech perception was associated with the three phonological processing abilities, and these phonological skills together predicted word reading. McBride‐Chang (1996) hypothesized that the association between speech perception and word reading was mediated via phonological awareness.

McBride‐Chang et al. (1997) assessed the development of phoneme awareness from Kindergarten to Grade 1 in 142 children tested four times at five‐monthly intervals from age 5 years. There were moderate correlations between speech perception and phoneme awareness both concurrently and longitudinally, and measures of non‐verbal cognitive ability, verbal memory and speech perception at age 5 years together predicted the growth of phoneme awareness and its final level (although in these analyses initial level of phonological awareness was not controlled). Poor speech perception at age 5 years was associated with poor reading at later stages, but speech perception was not a significant predictor of reading when phoneme awareness and letter knowledge were controlled.

Zhang and McBride‐Chang (2014) extended this work to investigate the concurrent predictors of Chinese (L1) and English (L2) reading in 7‐ to 9‐year‐old children using measures of auditory sensitivity, speech perception (segmental and suprasegmental), phonological awareness, RAN, verbal short‐term memory and morphological awareness (the phonological and morphological measures were in Cantonese). Speech perception had both direct and indirect effects on reading in English. The effect of segmental speech perception (categorical perception of consonants) on reading was mediated by phonological awareness, RAN, verbal short‐term memory and morphological awareness with a strong indirect path via phonological awareness. In addition, the direct path from suprasegmental perception (categorical perception of lexical tone) was significant. The finding of an indirect path from speech perception through phonological awareness to reading warrants replication in a monolingual English sample using a longitudinal design.

In short, given the various methodological issues surrounding previous studies of speech perception in dyslexia using the categorical speech perception task, and the dearth of longitudinal data, there is need for further research investigating the hypothesis of a causal chain from speech perception through phonological awareness to reading. If poor speech perception is a cause of poor reading, then we would expect it to predict reading measured as a continuous dimension and to be associated with poor reading/dyslexia defined categorically.

The present study uses data from a longitudinal study of children at high risk of dyslexia either because they had an affected parent or because they experienced a preschool language impairment placing them at risk of Developmental Language Disorder. The present dataset includes measures of speech perception, language, phoneme awareness, RAN, reading and attention when the children were aged 5½ and 6½ years. We also used outcomes at age 8 years to investigate the association of speech perceptual deficits with dyslexia and developmental language disorder.

We tested the following hypotheses:

1. Speech perception at 5½ years will predict phoneme awareness and RAN concurrently.
2. Speech perception at 5½ years will predict reading at 6½ years indirectly, via phoneme awareness and RAN, both for ‘at‐risk’ and control groups.
3. Language skills will be associated concurrently with speech perception ability.
4. Speech perception deficits will be associated with poor language outcomes at age 8 years rather than poor reading (dyslexia) per se, consistent with the findings of Joanisse and colleagues (Joanisse et al., 2000; Robertson et al., 2009).

## 2 METHOD

### 2.1 Participants

Children participating in this study were from the Wellcome Language and Reading Project. Ethical permission for the project was granted by the Psychology Department, University of York, and the NHS Research Ethics Committee; and informed written consent was obtained from the children's parents. Children entering the overarching longitudinal study were either at family risk (FR) of developing dyslexia, had a preschool language impairment (LI) or were typically developing (TD) (see Nash, Hulme, Gooch, & Snowling, 2013 for further details). Children were assessed at approximately annual intervals: t1 (3–4 years), t2 (4–5 years), t3 (5–6 years), t4 (6–7 years), t5 (8–9 years). Here we report data from 237 children at three time points: t3, t4 and t5.

Children were designated FR if they had a parent or sibling who could be classified as dyslexic. At t1 (age 3½), LI status was confirmed if their scores fell below criterion on at least two out of four language tests. The criterion was set as a scaled score of 7 or below for three subtests from the Clinical Evaluation of Language Fundamentals (CELF‐Preschool 2 UK; Wiig, Secord, & Semel, 2006): Basic Concepts, Expressive Vocabulary, Sentence Structure; and on the Test of Early Grammatical Impairment (TEGI; Rice & Wexler, 2001) the criterion was failure on the screener.

At t5 (age 8 years), we classified participants as fulfilling criteria for Dyslexia and/or Developmental Language Disorder (Bishop, Snowling, Thompson, & Greenhalgh, 2016) for comparison with controls. Dyslexia was assessed by performance on the Single Word Reading Test (SWRT‐6‐16; Foster, 2007) and the Wechsler Individual Achievement Test, Spelling Test (WIAT II; Psychological Corporation, 2005). Dyslexia was defined as falling at >1.5*SD* below the TD mean of 107 of this composite (a score of 89 or less). DLD was defined by performance on a composite language measure comprising the age‐standardized scores from Expressive Vocabulary (CELF‐4, UK; Wiig et al., 2006), Test for Reception of Grammar (TROG‐II; Bishop, 2003) and Formulated Sentences (CELF 4). DLD was defined as falling −1*SD* below the control mean. Using these criteria, of the 234 children remaining in the sample at age 8 years, 21 were identified as having dyslexia only, 38 as having DLD and 29 as comorbid dyslexia+DLD; 146 children were free of developmental disorder (of these 64 were recruited as controls, not at‐risk, the others were from one of the at‐risk groups).

### 2.2 Tests

Each child was administered a comprehensive battery of cognitive, language and literacy tests at each time point. Here we only report details of the measures which were used in the present analyses and those used to classify children into subgroups.

#### 2.2.1 Nonverbal ability

At t1, nonverbal ability was measured using two subtests from the Wechsler Preschool and Primary Scale of Intelligence (WPPSI‐III; Wechsler, 2003): Block Design and Object Assembly.

#### 2.2.2 Language measures

##### Vocabulary

Expressive vocabulary was assessed using the Expressive Vocabulary subtest at t1 (CELF‐Preschool 2 UK*;* Wiig et al., 2006) and t5 (CELF 4; Semel, Wiig, & Secord, 2003). At t5, the test was extended with eight items from the Expressive One Word Picture Vocabulary Test (Brownell, 2000) to guard against ceiling effects.

Receptive language was assessed at t1 using the Basic Concepts subtest (CELF‐Preschool 2 UK), and at t5 with the Receptive One Word Picture Vocabulary Test (Brownell, 2000).

##### Grammar

At t1 and t3, receptive grammar was assessed using the Sentence Structure subtest (CELF 4). Inflectional morphology at t1 was measured via two subtests of the TEGI (Rice & Wexler, 2001): the third person and past tense probes.

At t3, t4 and t5, sentence repetition was assessed using an experimental sentence imitation task (ESIT). This measure, designed for the present study, required the child to repeat 20 sentences: 10 (5 long/5 short) containing transitive verbs and 10 (5 long/5 short) containing ditransitive verbs.

At t5, receptive grammar was assessed using the Test for Reception of Grammar (TROG‐II; Bishop, 2003). Expressive grammar was measured using the Formulated Sentences subtest (CELF 4).

#### 2.2.3 Phonological measures

##### Phoneme awareness

At t3, the Phoneme Deletion subtest from the YARC (Hulme et al., 2009) was administered. In this test, the child hears a word, repeats it and then says it dropping a specified phoneme (e.g. ‘without the /b/’) (12 items). At t5, items were added to extend the test (five words with picture support and seven nonwords without picture support) to guard against ceiling effects.

##### Rapid naming

At t3, the RAN Objects task was given. Children name an 8 × 5 array of 40 stimuli as quickly as possible for two trials each. RAN rate is calculated as the mean number per second. RAN rate was calculated across items in each of two halves of the task.

##### Reading skills

Single word reading was measured at t3 and t4 using the Early Word Reading Test (Hulme et al., 2009) and the Single Word Reading Test (SWRT; Foster, 2007). The SWRT was also used to assess reading at t5.

The Spelling subtest of the Wechsler Individual Achievement Test (WIAT‐II; Wechsler, 2005) was administered at t5.

##### Categorical perception

Speech perception was measured at t3 (5½ years) using a test of categorical perception devised by Hazan et al. (2009; see also Hazan, Messaoud‐Galusi, & Rosen, 2013). In the literature, some 50% of studies use voicing stimuli (Noordenbos & Serniclaes, 2015); we chose the voicing contrast ‘bee’–’pea’ because it had been used recently in a study of children with dyslexia by Messaoud et al. (2011) and offered the possibility of replication. Children heard a token that varied along a synthetic ‘bee–pea’ continuum and were shown two pictures, one of a ‘bee’, one a ‘pea’. Their task was to decide if the token they heard was ‘bee’ or ‘pea’ and to touch the corresponding picture.

Stimuli were generated by copy synthesis of a natural [bi] token recorded from a female native British English speaker through use of the cascade branch of the Klatt (1980) synthesizer. The continuum was generated by delaying the onset of voicing while concurrently increasing the aspiration duration to obtain stimuli differing in voice onset time (VOT) ranging from 0 ms at the /bi / end to 60 ms at the /pi/ end of the continuum (for a full description, see Hazan et al., 2009). The task used a one‐interval, two‐alternative adaptive forced‐choice procedure. Two independent adaptive tracks were used. The two tracks, which operated under identical rules but started at opposite ends of the continuum, were designed to track 71% and 29% of ‘bee’ responses using a two‐down/one‐up rule (Levitt, 1971). On any particular trial, the choice of track was at random. The task ended after seven reversals on each track (with step sizes decreasing over the first three reversals) or a maximum of 50 trials. Catch trials (continuum endpoints) were randomly interspersed 20% of the time so that participants would not hear an uninterrupted sequence of ambiguous stimuli. Performance on the interspersed endpoints also provided a measure of response consistency throughout the task. Since catch trials were accurately identified at the start of the task by every listener, a reduction in correct identifications of catch trials as the test proceeded would indicate lapses in attention.

We used logistic regression to fit a sigmoid curve to the data for each participant, from which three measures were extracted: (1) the slope of the identification function that provides information on labelling consistency for the entire function, (2) the t‐slope that provides information on labelling consistency for the test items only (excluding the randomly interspersed catch trials) and (3) the phoneme boundary (based on the test trials only) that indicates the point along the VOT continuum that is equally labelled as /b/ or /p/. The slope and t‐slope values were skewed in our data so were log transformed for analysis. In the between‐groups analyses we also analysed the catch trials to provide a measure of the level of attention maintained through the task.

##### Attention

To complement data from the catch trials, we formed a factor score from ratings made by examiners of children's attention during three tasks administered during the same session in which the categorical perception task was administered. Ratings were made on a 5‐point scale during two executive function tasks (Auditory Continuous Performance Test; Simple RT task) and an auditory discrimination task (1= poor attention; 5 = excellent attention).

### 2.3 Procedure

At ages 5½ (t3), 6½ (t4) and 8 (t5) years, the children were tested either at school or at home in a single testing session with breaks as appropriate. At age 5½ years, they completed the categorical perception task twice, at the beginning and end of the testing session.

## 3 RESULTS

First, we wished to assess the patterns of longitudinal predictive relationships between measures of speech perception, language, phonological skills, and later literacy skills in children at‐risk of literacy difficulties (pooling across family risk and preschool language impairment). Second, we classified children at 8 years as having (1) Dyslexia, (2) DLD, (3) comorbid dyslexia with DLD or as typically developing (TD) controls and assessed differences amongst these groups in speech perception at 5½ years in retrospective analyses.

### 3.1 Relationships between speech perception, phoneme awareness, language and reading

Initial exploration of the data indicated that the pattern of relationships between variables was very similar in the children at family risk of dyslexia and those referred because of concerns about preschool language difficulties. Because of this, we combined these two at‐risk groups into one.

The means, standard deviations and reliabilities for all variables for the at‐risk and control groups are shown in Table 1. As would be expected, the control group generally performed better than the at‐risk group on the language and literacy measures.

**Table 1:** Means (and *SD*s) for language, phonological, reading and speech perception measures at t3 (5½ years) and t4 (6½ years) for control and at‐risk groups

		Reliability	Control (*N* = 74)		At‐Risk (*N* = 163)		d [95% CI]
Vocabulary^1^	*t3*	0.84	31.69	6.01	25.01	9.20	0.80 [0.52, 1.08]
Sentence Repetition^2^	*t3*	0.78	10.53	4.21	7.10	4.43	0.52 [0.24, 0.79]
Phoneme Awareness^3^	*t3*	0.93	7.76	2.24	5.96	2.78	0.69 [0.40, 0.97]
RAN Objects^4^	*t3*	0.70^9^	0.93	0.19	0.81	0.20	0.64 [0.36, 0.93]
Early Reading^5^	*t3*	0.98	20.15	8.04	14.09	8.67	0.71 [0.43, 1.00]
	*t4*		27.45	4.87	21.87	8.24	0.74 [0.46, 1.02]
Word Reading^6^	*t3*	0.98	15.53	13.53	8.05	9.35	0.69 [0.41, 0.97]
	*t4*		27.49	10.41	18.06	12.72	0.78 [0.50, 1.07]
NVIQ 7	*t1*		115.61	13.93	104.98	14.18	0.75 [0.46,1.04]
T slope‐1^8^	*t3*	0.45^9^	0.33	0.25	0.22	0.19	0.50 [0.22, 0.78]
T slope‐2^8^			0.38	0.27	0.26	0.22	0.54 [0.25, 0.82]
CatchTrials‐1	*t3*	0.34^9^	0.92	0.10	0.87	0.14	0.40 [0.12, 0.67]
CatchTrials‐2			0.90	0.11	0.86	0.13	0.33[0.05, 0.62]

Notes

^1^CELF Expressive Vocabulary Raw Score; ^2^Experimental Sentence Repetition Test; max = 20; ^3^YARC Phoneme Deletion Raw Score; ^4^RAN Objects Items/sec ^5^YARC Early Word Reading; ^6^Single Word Reading Test; ^7^Standard Score; ^8^logit/ms 9correlation between two runs (test–retest). Reliabilities are Cronbach's alpha unless otherwise specified.

Correlations between the language, reading and categorical speech perception measures for the at‐risk group and the control group are shown in Table 2. At t3 (age 5½ years) we assessed four constructs: Speech Perception (the slope of the categorical perception function assessed on two separate occasions (T‐slope, log transformed), Language (CELF Expressive Vocabulary and Experimental Sentence Imitation Test), Phoneme Awareness (phoneme deletion), Rapid Naming (Objects) and Reading (two measures of single word reading ability: Early Word Reading Test (Hulme et al., 2009) and Single Word Reading Test (Foster, 2007). All correlations were significant (*p* < 0.001) with the exception of some of the correlations with catch trials. These were generally higher for the at‐risk than for the control group. At t4 (age 6½) we used the same measures of reading to assess longitudinal relationships.

**Table 2:** Correlations between language and reading‐related measures at age 5½ (t3) and 6½ (t4) years (control above diagonal, at‐risk group below diagonal) with measures of speech perception

	Vocab^1^ t3	Sent Rep^2^ t3	Phon Aw^3^ t3	RAN^4^ t3	Early Read^6^ t3	Word Read^7^ t3	Early Read^6^ t4	Word Read^7^ t4	Tslope 1 t3	Tslope 2 t3	Catch Trials 1 t3	Catch Trials 2 t3
Vocab^1^ t3	–	0.52	0.36	0.21	0.47	0.45	0.36	0.47	0.44	0.29	0.17	0.09
Sent Rep^2^ t3	0.52	–	0.42	0.17	0.36	0.35	0.34	0.39	0.40	0.21	0.19	0.15
Phon Aw^3^ t3	0.42	0.49	–	0.39	0.55	0.52	0.50	0.57	0.43	0.40	0.15	0.02
RAN^4^ t3	0.42	0.45	0.56	–	0.40	0.35	0.50	0.45	0.19	0.32	0.28	0.20
Early Read^6^ t3	0.50	0.50	0.71	0.58	–	0.92	0.76	0.82	0.37	0.42	0.11	0.23
Word Read^7^ t3	0.45	0.41	0.67	0.55	0.90	–	0.62	0.79	0.27	0.30	0.16	0.23
Early Read^6^ t4	0.47	0.50	0.67	0.48	0.80	0.64	–	0.88	0.48	0.52	0.28	0.32
Word Read^7^ t4	0.47	0.51	0.67	0.52	0.89	0.84	0.82	–	0.41	0.45	0.27	0.24
Tslope 1 t3	0.33	0.32	0.21	0.32	0.26	0.21	0.31	0.27	–	0.61	0.27	0.20
Tslope 2 t3	0.29	0.30	0.38	0.40	0.33	0.30	0.29	0.32	0.31	–	0.04	0.21
Catch Trials 1 t3	0.27	0.22	0.25	0.20	0.38	0.32	0.28	0.36	0.34	0.26	–	0.36
Catch Trials 2 t3	0.14	0.13	0.26	0.19	0.25	0.17	0.27	0.27	0.36	0.34	0.29	–

Notes

^1^CELF Expressive Vocabulary; ^2^Experimental Sentence Repetition Test; ^3^YARC Phoneme Deletion; ^4^RAN Objects; ^5^Attention (latent factor score); ^6^YARC Early Word Reading; ^7^Single Word Reading Test.

Our principal interest was to trace possible causal influences from variations in speech perception skills measured in the early stages of formal literacy instruction to variations in later reading skills. A two‐group (at‐risk vs. typically developing) structural equation model (SEM) was estimated to assess the predictors of reading skills in Mplus 8 (Muthén & Muthén, 1998) using maximum likelihood estimation. There were small proportions of missing data (minimum proportion of data present for all covariances was 0.926 for the control and 0.932 for at‐risk groups). Missing data were handled with Full Information Maximum Likelihood estimation (FIML). In this model, a speech perception latent variable at age 5½ years is defined by an estimate of the slope of the psychometric function derived from two separate runs of the categorical speech perception task (log transformed). Both the factor loadings and residuals of the observed speech perception variables were fixed to be equal as the two runs were considered to be parallel. Reading in this case is measured by a latent variable defined by one indicator (summed scores from the two single word reading tests) with a reliability of 0.98 (taken from earlier reliability estimates of these variables). This approach was taken because of problems with the distributions of the two reading measures (EWR showed strong trends towards a ceiling effect, and there were indications that although these two measures were very highly correlated, there was both a linear and a quadratic component to the relationship between them). As we had only one indicator of phoneme awareness, the residual variance of the observed phoneme deletion measure was fixed in line with the reliabilities of the measures estimated at age 5½ years (alpha = 0.93). A latent RAN variable was estimated by the two runs of the RAN objects test (with the factor loadings fixed to be equal). A latent attention variable was estimated by the three attention indicators and a latent language variable was estimated by CELF Expressive Vocabulary and the Experimental Sentence Imitation Test. A comparison between a confirmatory factor analysis (CFA) with and without the factor loadings and the intercepts of these latent variables fixed to be equal across groups showed that there was full scalar invariance (factor loadings and intercepts) of the measurement model including all variables over groups, Δχ^2^ = 7.546 (9), *p* = 0.581.

**Figure 1:**
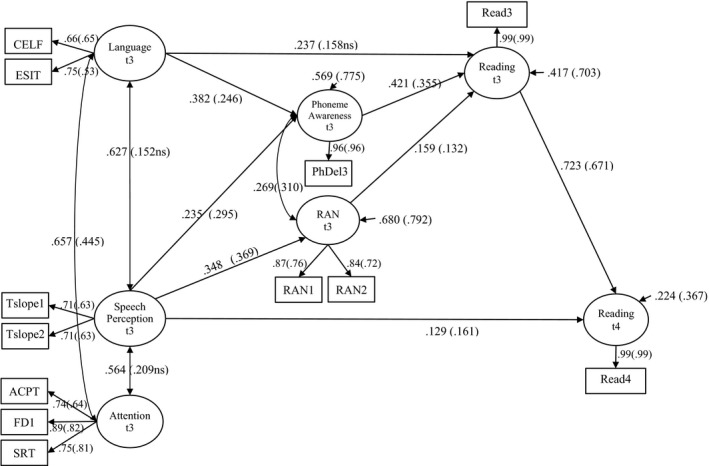
Longitudinal model showing predictive relationships between speech perception at age 5½ years and reading at 6½ years (standardized coefficients for the at‐risk group outside the brackets and standardized coefficients for the control group inside the brackets). All coefficients are significant *p* < 0.05 with the exception of the one marked as *ns* (not significant). Ellipses represent latent variables, rectangles represent observed variables. One‐headed arrows represent regressions, factor loadings (from latent variables to its observed indicators) or residual variances (from a number). Double‐headed arrows represent correlations

In the model shown in Figure 1, we regressed phoneme awareness and RAN on language, speech perception and attention, all measured at t3 (5½ years). Further, reading at 5½ years was regressed on all other variables measured at the same age (language, speech perception, attention, phoneme awareness and RAN) and the same was the case for reading at 6½ years (t4) with the addition of controlling for the autoregressor (reading at 5½ years, t3).

In the final model, all direct unstandardized paths were fixed to be equal across groups with the exception of the autoregressor (Reading at 5½ years → Reading at 6½ years) as this varied significantly across the two groups. The model is shown in Figure 1 with the non‐significant paths not shown for the sake of simplicity (although they were retained in the model). As can be seen, speech perception and reading at age t3 (5½) years were unique predictors of reading at age t4 (6½ years). Language (not significant in the control group), phoneme awareness and RAN at t3 (5½ years) contributed uniquely to reading concurrently. Speech perception and language at t3 (5½ years) were unique concurrent predictors of phoneme awareness. Speech perception at age 5½ years was a unique concurrent predictor of RAN. However, there were no significant indirect effects of speech perception and language to reading at age 5½ years or 6½ years (to test this, we bootstrapped the confidence intervals of the indirect effect to take account of the non‐normal distributions often found in indirect effects; Biesanz, Falk, & Savalei, 2010). The fit of this model with all paths estimated as in Figure 1 is excellent (χ^2^ = 104.753 (101), *p* = 0.378, RMSEA = 0.018 (90% CI = 0.000–0.052), CFI = 0.997, TLI = 0.996). To see if the lack of indirect effects depended on our control for attention skills we also estimated the same indirect effects in a trimmed version of our model in which all insignificant regression paths were deleted (see Table 3). In this trimmed model, we found significant total indirect effects of speech perception on reading at age 5½ and 6½ years in both groups. However, the only specific indirect effect was via RAN (speech perception → RAN → reading); the effect via phoneme awareness (speech perception → phoneme awareness → reading) was far from significant. Similarly, the indirect effect of language on reading through phoneme awareness was not significant (see Table 3). In the trimmed model, the path from language to phoneme awareness at 5½ years was also significant in the control group only. This model did not have a worse fit than the untrimmed model, Δχ^2^ = 10.813 (9), *p* = 0.289

**Table 3:** Standardized indirect effects in the trimmed model from speech perception and language to reading at age 5½ years and 6½ years in the at‐risk and in the control groups

	At‐risk		Control	
	β	95% CI	β	95% CI
Total indirect effect on Read t3 from *Speech Perception*	0.239	0.033,0.506	0.209	0.033,0.443
Indirect effects on Read t3 through:				
Phoneme Deletion	0.134	−0.049,0.411	0.117	−0.049,0.335
RAN	0.105	0.018,0.188	0.092	0.014,0.180
Total indirect effect on Read t4 from *Speech Perception*	0.184	0.025,0.372	0.148	0.022,0.296
Indirect effects on Read t4 through:				
Phoneme Deletion	0.103	−0.038,0.311	0.083	−0.034,0.229
RAN	0.081	0.013,0.144	0.065	0.010,0.132
Indirect effects on Read t3 from Language through:				
Phoneme Deletion	0.166	−0.022,0.362	0.105	−0.017,0.210
Indirect effects on Read t4 from Language through:				
Phoneme Deletion	0.128	−0.016,0.281	0.074	0.011,0.163

Note

The confidence intervals of the indirect effects are bootstrapped with 1,000 draws. Significant coefficients in bold.

The pattern of correlations between the latent variables in the model is informative (see Table 4). As would be expected, most latent variables are positively correlated with each other. Both speech perception and phoneme awareness are moderate to strong longitudinal predictors of reading ability at age 6½ years and these two predictors are quite highly correlated with each other.

**Table 4:** Estimated correlations between the latent variables in Figure 1 (control above and ‘at‐risk’ below the diagonal)

	Reading_t3	Reading_t4	T_Slope_T3	Phoneme Awareness_t3	RAN_t3	Language _t3	Attention _t3
Reading_t3	–	0.768**	0.252*	0.497**	0.359**	0.345**	0.229**
Reading_t4	0.867**	–	0.354**	0.485**	0.355**	0.341**	0.170
SP_Slope_t3	0.545**	0.579**	–	0.315**	0.401**	0.152	0.209
Phoneme Awareness_t3	0.702**	0.672**	0.548**	–	0.442**	0.384**	0.298**
RAN_t3	0.545**	0.518**	0.522**	0.529**	–	0.275*	0.169
Language_t3	0.635**	0.629**	0.627**	0.615**	0.497**	–	0.445*
Attention_t3	0.497**	0.454**	0.564**	0.515**	0.375**	0.657**	–

Note

*p > 0.05; **p > 0.01.

### 3.2 Deficits in speech perception in dyslexia and developmental language disorder

Table 5 shows performance on the categorical perception task for children at age 8 years who were classified as dyslexic, DLD or with comorbid dyslexia and DLD and typically developing (TD) controls without either disorder.

**Table 5:** Means (and *SD*s) as a function of diagnostic group at age 8 years on measures of categorical perception at 5½ years and NVIQ

		Outcome Group				Effect Size (d)		
		TD‐Control^3^ (*N* = 54)	Dyslexia (*N* = 16)	Developmental Language Disorder (DLD) (*N* = 29)	Dyslexia + DLD (*N* = 24)	TD‐Dys	TD‐DLD	TD‐Dys+DLD
Categorical Perception	Average T‐slope^1^	−0.57 (0.28)	−0. 79 (0.22)	−1.02 (0.49)	−1.09 (0.40)	0.83 [0.25, 1.40]	1.23 [0.74, 1.72]	1.64 [1.09, 2.19]
	Proportion of Catch Trials Correct^1^	0.91 (0.08)	0.86 (0.08)	0.78 (0.14)	0.82 (0.11)	0.63 [0.06, 1.19]	1.24 [0.75, 1.73]	1.00 [0.49, 1.50]
NV IQ 2		111.96 (14.1)	106.95 (14.2)	100.08 (14.4)	101.32 (11.6)	0.35 [−0.13, 0.84]	0.84 [0.46, 1.21]	0.77 [0.34, 1.21]

Notes

^1^Averaged across two runs; ^2^Standard score at 3½ years; ^3^Participants from Control group (at t1) with no disorder at t5.

**Figure 2:**
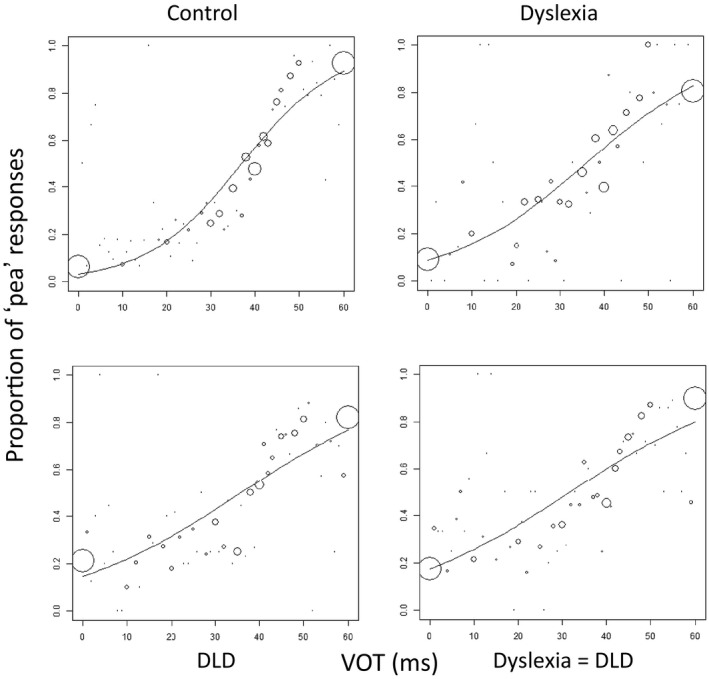
Identification functions for the ‘pea’–’bee’ continuum for the Control Group (upper left), the Dyslexia Group (upper Right), the DLD group (lower left) and the Dyslexia + DLD group (lower right). The circles represent the number of ‘pea’ responses along the voice onset time (VOT) continuum aggregated across data in the group. The size of the circles is proportional to the number of presentations at a given VOT (0–60 ms). The solid line is the result from a logistic regression on data from all trials

Effect sizes (Cohen's *d*) are shown for the comparisons of T‐slope scores and for performance on catch trials in the categorical perception task between the typically developing (TD) controls and each of the groups with disorders. The data show a stepwise pattern (TD>Dyslexia>DLD> Dyslexia + DLD): typically developing children showed better categorical perception as evidenced by the T‐slope of the identification function than the groups with dyslexia, DLD and the comorbid group. This can also be seen in Figure 2 which illustrates the identification function for the four groups for the ‘pea’–’bee’ continuum: the slopes are shallower for the two DLD groups than for the group with dyslexia and the TD‐control group.

**Figure 3:**
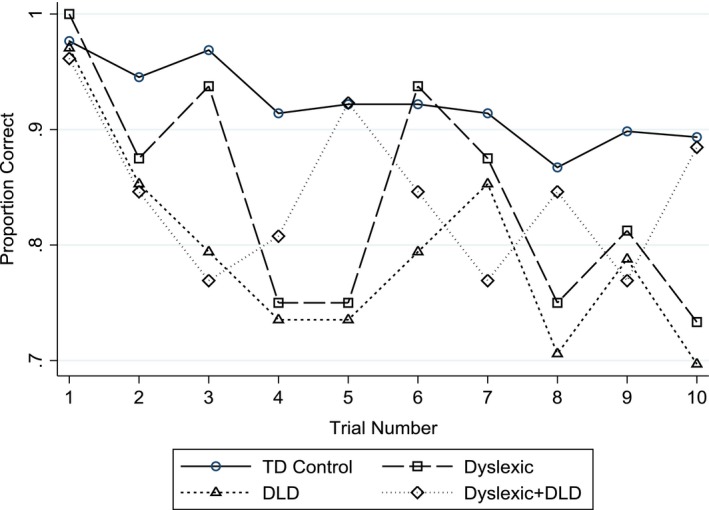
Performance across catch‐trials for Dyslexia, DLD, Dyslexia + DLD and TD‐control groups (proportion of the group responding correctly per trial)

Figure 3 shows the performance on the 10 ‘easy’ catch‐trials for the four groups in terms of the proportion of the group responding correctly on each trial. Messaoud et al. (2011) regarded such data as a measure of attention although it has also been argued that errors on ‘easy’ trials may reflect perceptual processing effects. The TD controls show better performance than the three ‘clinical’ groups overall. It is clear that performance across trials is highly variable, particularly in the clinical groups.

An individual growth curve model with random intercepts but fixed slopes across children showed that there was a significant linear effect of trial (unstandardized slope = −0.0095 [95% CI −0.0157, −0.0033], *z*= −3.00. *p* = 0.003). There was no significant quadratic component. This linear effect confirms that performance on the catch trials declines across trials consistent with a waning of attention to the task. However, there was no significant interaction between slope and group giving no evidence for a differential decline in attention across groups. Collapsing across trials the three clinical groups all showed poorer performance than the controls (but none of the clinical groups differed significantly from each other).

A series of ANOVAs showed that, while there were overall group differences in T‐slope (*F*(3, 119) = 16.55, *p* < 0.001) and on accuracy on the catch trials (*F*(3, 119) = 10.91, *p* < 0.001), the group with dyslexia did not differ significantly from TD controls on either measure, whereas the two groups with DLD performed significantly worse on both measures than controls. The effect size of the difference in performance between the dyslexic and control group is large on the categorical perception task (*d* = 0.83) and medium on catch trials (*d* = 0.63); it is very large for both DLD groups on each measure. It is notable given the association of both language and attention with speech perception in our model, and the particularly strong path weights for the at‐risk group, that the speech perception deficit is more strongly associated with DLD than with dyslexia.

## 4 DISCUSSION

We used longitudinal data from a large high‐risk sample of 5½–6½‐year‐old children to investigate the hypothesis that poor speech perception is a causal risk factor for dyslexia. If this were true, we would expect a predictive relationship between performance on categorical speech perception at 5½ years and reading one year later, and an association between poor speech perception and a subsequent dyslexia outcome at age 8 years.

We found weak support for the causal hypothesis that speech perception influences the development of reading indirectly via its effects on either phoneme awareness or RAN (speech perception → phoneme awareness and RAN → reading). Although speech perception predicted phonological skills (phoneme awareness and RAN) concurrently and these, in turn, predicted reading at the same point in time, the indirect effects via phoneme awareness were not significant, although the indirect effects via RAN to reading were. The finding of a relationship between speech perception and RAN was not anticipated and is hard to explain. It might be that good speech perceptual skills depend in part on well‐specified phonological representations, which in turn play a key role in limiting RAN. This issue clearly deserves further study.

Longitudinally there was high stability between reading at 5½ years and 6½ years and even after the autoregressor (reading at 5½ years) was controlled, speech perception was a direct predictor of reading at age 6½ years. In short, the relationship between speech perception at 5½ years and reading a year later was direct. This held true for both at‐risk and control groups. The finding that speech perception longitudinally has a direct effect on reading a year later is somewhat surprising, and suggests that our measure of speech perception relates to reading in ways that are separable from its effects on phoneme awareness.

We also found that speech perception was correlated with language and with ratings of attention. Correlations were stronger for the at‐risk than control group (χ^2^ (1) = 4.235, *p* = 0.040 attention‐speech perception; χ^2^ (1) = 5.680, *p* = 0.017 language‐speech perception) suggesting that language and attention may influence performance on the speech perception task more strongly in the at‐risk group. Although the categorical speech perception task is complex and performance may be influenced by attentional and language skills, the direct effects of speech perception on reading cannot be explained by variations in attention or language skills since these were controlled in the model.

Finally, it is worth noting that language skills predicted reading skills directly and via phoneme awareness (although both of these effects were stronger in the at‐risk than the control group). This pattern of stronger predictions from language to reading skills in the at‐risk group suggests that broader oral language skills may place constraints on the development of decoding skills, particularly amongst children with language weaknesses (Hulme, Nash, Gooch, Lervåg, & Snowling, 2015). Language skills were also strongly correlated with RAN. This is not unexpected, and naming objects appears to depend at least in part on semantic knowledge (Lambon‐Ralph, Patterson, & Hodges, 1997).

The former analyses examined reading and language as continuous dimensions. We next turned to examine whether speech perception deficits were associated with a poor language outcome at t5 (DLD) rather than poor reading (dyslexia), as previously reported by Joanisse et al. (2000). Our findings showed that typically developing children performed better on the categorical speech perception task than children with reading and/or language disorders. While the group impairment for dyslexia was smaller than for children with DLD, consistent with Robertson et al. (2009), the effect size for differences with controls was large. In interpreting these findings, it should be noted, as argued by Rosen and colleagues (Messaoud et al., 2011; Rosen, 2003), that we found poorer performance in the categorical perception task to be associated with poorer attention (see correlations in Table 4), as well as with poorer performance on easy catch trials, with considerable variability across these trials as the task progressed. It is therefore difficult to interpret poor performance on the categorical perception test in children with dyslexia or DLD that may reflect perceptual or attentional difficulties or both. Nonetheless, a problem for the view that poor speech perception is a cause of poor reading comes from the finding that children in the ‘non‐dyslexic’ DLD group also showed deficits on the categorical perception task.

Although we found that deficits in speech perception are associated with both poor reading and poor language skills, the causal relationships remain unclear. Categorical speech perception tasks require both perceptual and decision‐making skills (Treisman, 1999). Many children with dyslexia have comorbid language problems (McArthur, Hogben, Edwards, Heath, & Mengler, 2000) and problems of attention control are strongly associated with preschool language difficulties (Gooch, Hulme, Nash, & Snowling, 2014). Such comorbidities may go some way toward explaining the occurrence of speech perception deficits in dyslexia, at least when measured using the categorical perception task. Future research testing developmental models of dyslexia that trace its origins to infancy (Goswami, 2015; Zhang & McBride‐Chang, 2010) will need to consider how related processes, such as attentional skills, contribute to the developmental pathways that lead to reading and its disorders. An important finding of the present study is that, while speech perception is a predictor of reading, speech perception deficits are evident in children with DLD regardless of whether or not they are dyslexic. Furthermore, those deficits are more marked in children with DLD than in children with dyslexia in the absence of a language impairment. It is important for future studies to clarify both the specific and general effects of poor speech perception on development if we are to understand the heterogeneity of neurodevelopmental disorders.

In summary, we believe that this is the largest study to assess the relationship between speech perception (measured at the start of formal reading instruction) and later reading. Our findings provide at best limited evidence for the view that speech perception is causally related to learning to read. The evidence here, as in most behavioural studies, relies on a categorical speech task that also taps factors extraneous to speech perception. From our subgroup analyses, problems on the categorical speech perception task are more strongly associated with language deficits than reading problems; since such children also typically experience problems of attention and executive control (Henry, Messer, & Nash, 2012), these may cause particular problems for them on complex perceptual tasks such as categorical perception tasks.
